# Achalasia alters physiological networks depending on sex

**DOI:** 10.1038/s41598-024-52273-3

**Published:** 2024-01-24

**Authors:** Janette Furuzawa-Carballeda, Antonio Barajas-Martínez, Paola V. Olguín-Rodríguez, Elizabeth Ibarra-Coronado, Ruben Fossion, Enrique Coss-Adame, Miguel A. Valdovinos, Gonzalo Torres-Villalobos, Ana Leonor Rivera

**Affiliations:** 1 Department of Immunology and Rheumatology, Instituto Nacional de Ciencias Médicas y Nutrición Salvador Zubirán, 14080 Mexico, Mexico; 2 Centro de Ciencias de la Complejidad, Universidad Nacional Autónoma de México, 14060 Mexico, Mexico; 3 Instituto de Ciencias Nucleares, Universidad Nacional Autónoma de México, 14060 Mexico, Mexico; 4 Departamento de Fisiología, Facultad de Medicina, Universidad Nacional Autónoma de México, 14060 Mexico, Mexico; 5 Department of Gastroenterology, Instituto Nacional de Ciencias Médicas y Nutrición Salvador Zubirán, 14080 Mexico, Mexico; 6 Departments of Surgery and Experimental Surgery, Instituto Nacional de Ciencias Médicas y Nutrición Salvador Zubirán, 14080 Mexico, Mexico

**Keywords:** Biomarkers, Diseases, Gastroenterology, Medical research

## Abstract

Achalasia is a rare esophageal motility disorder for which the etiology is not fully understood. Evidence suggests that autoimmune inflammatory infiltrates, possibly triggered by a viral infection, may lead to a degeneration of neurons within the myenteric plexus. While the infection is eventually resolved, genetically susceptible individuals may still be at risk of developing achalasia. This study aimed to determine whether immunological and physiological networks differ between male and female patients with achalasia. This cross-sectional study included 189 preoperative achalasia patients and 500 healthy blood donor volunteers. Demographic, clinical, laboratory, immunological, and tissue biomarkers were collected. Male and female participants were evaluated separately to determine the role of sex. Correlation matrices were constructed using bivariate relationships to generate complex inferential networks. These matrices were filtered based on their statistical significance to identify the most relevant relationships between variables. Network topology and node centrality were calculated using tools available in the R programming language. Previous occurrences of chickenpox, measles, and mumps infections have been proposed as potential risk factors for achalasia, with a stronger association observed in females. Principal component analysis (PCA) identified IL-22, Th2, and regulatory B lymphocytes as key variables contributing to the disease. The physiological network topology has the potential to inform whether a localized injury or illness is likely to produce systemic consequences and the resulting clinical presentation. Here we show that immunological involvement in achalasia appears localized in men because of their highly modular physiological network. In contrast, in women the disease becomes systemic because of their robust network with a larger number of inter-cluster linkages.

## Introduction

Achalasia is a primary esophageal motor disorder. It is a rare disease with an annual incidence of 1–5 cases and a prevalence of 7–32 cases per 100,000 individuals. Achalasia can occur at any age, but incidence and prevalence increase with age, and the mean age at diagnosis is between 30 and 60 years, without distinction of ethnicity. It is characterized by a failure to relax the lower esophageal sphincter (LES) in response to swallowing. It is accompanied by aperistalsis of the esophageal body, resulting in severe dysphagia, regurgitation, aspiration, chest pain, and weight loss^1^. Some studies have proposed that achalasia may be an autoimmune disease^2^, wherein the potential causal agent could be a neurotropic virus that chronically infects the myenteric plexus of the esophagus in genetically susceptible hosts, leading to the breakdown of immunological tolerance^3^. Proposed infectious agents include herpes simplex virus type 1 (HSV-1, a neurotropic virus with a preference for mucosal squamous epithelium, which persists in a latent state in neurons and induces a strong humoral and cellular immune response), varicella-zoster, measles, human papillomavirus, mumps, John Cunningham virus (commonly known as “JC”, a member of the polyomavirus family), and bornavirus (a related disease of psittacine birds that shows many similarities to achalasia)^3,4^. The susceptibility to develop achalasia has been associated with the HLA-DQ$\beta$1 alleles (HLA-DQ$\beta$1*03:04, HLA-DQ$\beta$1*05:03, HLA-DQ$\beta$1*06:01, and DQ$\beta$1*0602), HLA-DQ$\alpha$1 (HLA-DQ$\alpha$1*01:03), and HLA-DR$\beta$*127-9. In the mestizo-Mexican population, the HLA class II alleles HLA-DRB1*14:54:01 and DQB1*05:03:01, as well as the extended haplotype, are risk factors for achalasia^5^. In patients with achalasia, the LES muscle exhibits diverse inflammatory infiltrates (known as plexitis), primarily composed of lymphocytes and macrophages, with a lesser presence of polymorphonuclear cells. The characterization of the intraganglionic infiltrate shows a predominance of CD3+, CD4+ (Th22, Th17, Th2, and Th1), CD25+ and CD8+ T lymphocytes, as well as CD20+ B cells, eosinophils, and plasma cells located along the nerve bundles and around the ganglion cells^4,6–9^. Inflammatory infiltrates enriched for T and B cells generally predominate in tissues with advanced disease (> 10 years of symptomatic history)^6^. In achalasia, mast cell infiltration is associated with nitrergic nerves, S-100 positive cells, interstitial cells of Cajal, and neuronal degeneration^10–13^. An essential feature includes a reduction in the count of ganglion cells and Cajal cells, accompanied by fibrosis, which can account for as much as 21% of the overall tissue composition^14^. Furthermore, the LES muscle demonstrates an accelerated turnover of the extracellular matrix, driven by human gelatinase MMP-9 in its 92-kDa proform and 85-kDa active form. These enzymes contribute to the degradation of PNMA/Ta2 and GAD65, proteins expressed ectopically, which seem to be involved in the development of organ-specific autoimmune diseases, such as achalasia (referred to as REGA or ‘remnant epitopes generated autoimmunity’, mediated by cytokine-regulated tissue proteolysis)^14,15^. This proteolysis is associated with anti-GAD65 and anti-PNMA/Ta2 tissue-specific serum autoantibodies in patients with achalasia^3,14^. Proteomic studies and serum profile analysis of patients with achalasia have revealed significantly higher concentrations of pro-inflammatory cytokines (IL-22, IL-17, IL-4, IL-12, IL-6, IFN-$\gamma$, TNF-$\alpha$, etc.), C4B5, C3, cyclin-dependent kinase 5, $\alpha$2-macroglobulin, and anti-GAD65, anti-PNMA/Ta2, anti-triosephosphate isomerase, anti-carbonic anhydrase, and anti-creatinine kinase brain antibodies, when compared to those in healthy controls^16–21^. Finally, approximately 17–19% of patients with achalasia have autoimmune comorbidity, with 53% having hypothyroidism, and 68% having a family history of autoimmunity. When compared to individuals with gastroesophageal reflux disease (GERD), achalasia patients are 3.8 times more prone to autoimmune diseases (95% CI 1.47–9.83), 3.0 times more susceptible to thyroid disorders (95% CI 1.00–9.03), and 3.02 times more inclined to experience chronic inflammatory conditions (95% CI 1.65–6.20)^22^. However, achalasia is still not fully understood. Exploring the intricate interactions among various individual variables, including genes, proteins, physiological responses, and immunological reactions, to comprehend the emergence of system-wide behaviors is a current and ongoing challenge. Among various methods suitable for studying entire systems, the network physiology approach has yielded promising outcomes, especially by demonstrating the correlation between system-wide behavior, specifically physiological function, and network topology^23,24^. For instance, network approaches have previously been theorized to better elucidate the origin of autoimmune diseases^14^. This study aims to examine the main topological characteristics of a physiological network built from measurements of immunological biomarkers, including biopsy and in situ detection of neurons and immune cells, serum measurements of cytokines and autoantibodies, and flow cytometry classification of white blood cells. Employing this approach, we demonstrate how variations in physiological network topology can predict whether the development of achalasia tends to be localized or systemic in men and women, respectively.

## Results

### Childhood exanthems are common in patients with achalasia

A total of 189 patients with idiopathic achalasia were included in the study, comprising 116 (61%) females with a mean age of 42 ± 15 years and a disease duration of 24 months (range 1–150 months) at the time of assessment. The most frequent type of achalasia was type II (65%), followed by type I (29%) and type III (6%). The group of 500 healthy donors consisted of 315 females (comprising 63% of the total), with an average age of $36 \pm 12$ years. Clinical manifestations in patients with achalasia included dysphagia (98%), regurgitation (89%), heartburn (58%), and weight loss (87%) (Table 1). The prevalence of autoimmune comorbidity was 13% in achalasia patients, and 16% had a history of allergy or asthma. 64% and 31% of the patients had chickenpox or measles during childhood (Table 2). A systematic analysis was conducted to discern sex-based distinctions. Notably, there were no discernible variations in the clinical presentation of achalasia or its medical management between the two sexes. Differences emerged exclusively in hemoglobin concentration, tobacco smoke exposure, alcohol consumption, height, and body weight

**Table 1:** Demographic description of the database.

	Healthy donors	Achalasia (all)	Type I	Type II	Type III
	(n = 500)	(n = 189)	(n = 59)	(n = 123)	(n = 7)
Demographics					
Age (years) Mean ± SD	36 ± 12	42 ± 15	42 ± 15	42 ± 14	50 ± 17
Median	36	41	40.5	40	44
Range	18–63	18–79	18–79	18–77	26–77
Sex female, n (%)	315 (63)	116 (61)	26 (44)	85 (69)	5 (71)
Disease evolution (mo), Mean ± SD	NA	24 ± 27	27 ± 30	23 ± 27	18 ± 16
Median	NA	12	12	12	12
Range	NA	1–150	1–150	1–132	4–48
BMI (kg/m^2^) mean ± SD	NA	23 ± 5	23 ± 4	23 ± 5	27 ± 2
Median	NA	22	23.4	21.9	26.7
Range	NA	14.6–36.8	15.6–33.0	14.6–36.8	25.3–28.1
Overweight, n (%)	NA	58 (31)	15 (25)	39 (32)	4 (57)
Obesity, n (%)	NA	21 (11)	7 (12)	14 (11)	0 (0)
Symptoms					
Dysphagia, (n%)	NA	185 (98)	55 (93)	123 (100)	7 (100)
Regurgitation, (n%)	NA	168 (89)	51 (86)	112 (91)	5 (71)
Heartburn, (n%)	NA	109 (58)	29 (49)	79 (64)	1 (14)
Weight loss, (n%)	NA	164 (87)	47 (81)	111 (90)	6 (86)

**Table 2:** Personal antecedents and exposure.

	Healthy donors (n = 500)	Achalasia (all) (n = 189)	Type I (n = 59)	Type II (n = 123)	Type III (n = 7)
Personal antecedents					
Autoimmune disease, (n%)	NA	24 (13)	6 (10)	20 (16)	2 (29)
Allergy and asthma, (n%)	NA	30 (16)	10 (17)	20 (16)	0 (0)
Viral exanthematous disease					
Chickenpox, n (%)	NA	121 (64)	35 (59)	83 (67)	3 (43)
Rubella, n (%)	NA	18 (10)	4 (06)	14 (11)	0 (0)
Measles, n (%)	NA	58 (31)	22 (37)	34 (28)	2 (29)
Environmental exposure					
Tobacco smoke exposure, n (%)	NA	56 (30)	14 (24)	39 (32)	3 (43)
Alcohol consumption, n (%)	NA	65 (34)	22 (37)	41 (33)	2 (29)
Biomass smoke exposure, n (%)	NA	39 (21)	12 (20)	25 (20)	2 (29)

### Local and systemic immune activity in achalasia

**Table 3:** Complete blood count and C reactive protein.

	Healthy donors	Achalasia (all)	Type I	Type II	Type III
	(n = 500)	(n = 189)	(n = 59)	(n = 123)	(n = 7)
Laboratory variables					
Hemoglobin (g/dL) mean ± SD	15 ± 1	15 ± 2	15 ± 2	15 ± 2	15 ± 1
Median	15.3	14.8	15.2	14.5	15.7
Range	13.3–18.6	10.0–18.2	10.0–17.8	10.1–18.2	13.9–16.8
Platelets ($10^3$ cells/mL) mean ± SD	275 ± 52	231 ± 60	231 ± 53	234 ± 64	176 ± 46
Median	271	223	226.5	226	163
Range	144–460	128.0–427.0	145.0–392.0	135.0–427.0	128.0–258.0
Leukocytes ($10^3$ cells/mL) mean ± SD	7 ± 1	6 ± 2	6 ± 2	7 ± 2	6 ± 2
Median	6.9	6.2	6	6.5	5.5
Range	3.6–11.2	2.9–12.6	3.0–12.6	2.9–11.2	4.0–7.9
Lymphocytes (%) mean ± SD	33 ± 7	30 ± 9	31 ± 8	30 ± 10	33 ± 7
Median	32.6	30.1	31.2	29.5	32.3
Range	19.9–55.9	5.5–54.9	10.9–47.0	5.5–54.9	24.5–42.5
Monocytes (%) mean ± SD	7 ± 2	7 ± 2	7 ± 2	7 ± 2	7 ± 1
Median	7.6	7.1	7.6	6.8	6.6
Range	3.8–14.3	1.0–13.6	4.4–13.6	1.0–13.0	5.7–8.6
Neutrophils (%) mean ± SD	55 ± 8	59 ± 10	58 ± 10	60 ± 11	58 ± 7
Median	55.3	59.9	59	60	58.7
Range	37.9–71.5	30.0–92.4	37.0–78.4	30.0–92.4	47.1–68.0
Eosinophils (%) mean ± SD	2 ± 2	3 ± 2	3 ± 2	2 ± 2	1.6 ± 0.5
Median	2	2.3	2.3	1.6	1.6
Range	0.2–9.5	0.6–13.0	0.4–13.0	0.0–14.5	1.0–2.4
Basophils (%) mean ± SD	1.0 ± 0.3	0.6 ± 0.5	0.7 ± 0.6	0.6 ± 0.5	0.6 ± 0.4
Median	1	0.5	0.6	0.5	0.5
Range	0.2–2.1	0.0–5.0	0.0–3.7	0.0–5.0	0.3–1.3
NLR mean ± SD	1.8 ± 0.6	2 ± 2	2 ± 1	3 ± 2	1.9 ± 0.6
Median	1.7	2	1.9	2	1.8
Range	0.6–4.8	0.6–15.7	0.9–7.2	0.6–15.7	1.1–2.8
CRP (mg/dl) mean ± SD	NA	0.4 ± 0.7	0.5 ± 0.8	0.4 ± 0.7	0.4 ± 0.2
Median	NA	0.2	0.2	0.2	0.3
Range	NA	0.0–4.8	0.0–3.5	0.0–4.8	0.1–0.6

**Table 4:** Fluorescence-activated cell sorting.

	Healthy donors (n = 500)	Achalasia (all) (n = 189)	Type I (n = 59)	Type II (n = 123)	Type III (n = 7)
Circulating CD4+ T cell subpopulations					
CD4+/CD161-/IL-22+ (%) mean ± SD	2.3 ± 0.1	8.5 ± 0.4	9.4 ± 0.7	7.9 ± 0.5	10 ± 2
Median	2.51	7.74	9.05	6.98	11.7
Range	0.35–3.90	2.75–19.9	5.79–19.9	2.75–15.6	6.93–13.0
CD4+/CD161+/IL-17A+ (%) mean ± SD	2.0 ± 0.1	7.6 ± 0.3	7.9 ± 0.5	7.3 ± 0.3	9.8 ± 0.8
Median	2.12	7.61	8.11	7.55	10.5
Range	0.41–3.47	2.68–14.8	2.68–13.5	2.95–14.8	8.05–10.7
CD4+/CD25-/IL-4+ (%) mean ± SD	2.0 ± 0.1	7.1 ± 0.3	6.6 ± 0.4	7.5 ± 0.5	5.7 ± 1.7
Median	2.08	6.41	6.33	6.54	5.92
Range	0.23–3.93	2.72–17.1	2.81–10.2	2.90–17.1	2.72–8.58
CD4+/CD25-/IFN-$\gamma$+ (%) mean ± SD	2.0 ± 0.1	6.9 ± 0.3	6.8 ± 0.5	6.9 ± 0.4	7 ± 3
Median	1.91	6.17	6.31	6.12	4.9
Range	0.29–4.65	2.23–13.4	2.44–11.1	2.99–13.4	2.23–12.8
CD4+/CD25hi/Foxp3+ (%) mean ± SD	6.5 ± 0.3	9.2 ± 0.3	9.5 ± 0.4	8.9 ± 0.4	10 ± 1
Median	6.83	8.9	9.08	8.87	10.1
Range	1.34–9.20	1.36–15.6	6.26–13.5	1.36–15.6	8.07–12.3
CD19+/CD24+/CD38+/IL-10+ (%) mean ± SD	8 ± 3	10 ± 3	9 ± 3	10 ± 3	16 ± 1
Median	8.4	10.8	9.3	11	15.8
Range	2.0–13.7	4.1–17.4	4.1–14.0	4.1–15.9	14.6–17.4
CD123+/CD196+/IDO+ (%) mean ± SD	16.9 ± 0.6	37± 1	32 ± 2	38 ± 2	46 ± 2
Median	17.2	36	31.6	38.1	45.7
Range	6.79–28.1	16.8–62.0	21.5–47.4	16.8–62.0	43.1–49.3

**Figure 1:**
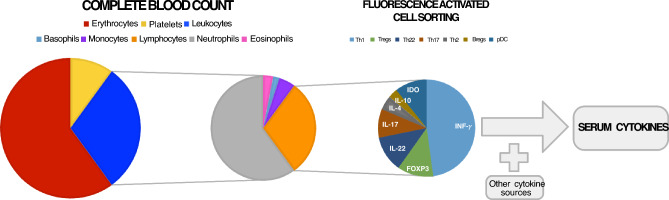
Circulating components of the immune system. Through an incremental approach we performed a complete blood count which differentiates leukocytes from lymphocytes, monocytes, basophils, eosinophils and neutrophils. Then the lymphocytes were furthed sorted by multiple fluorescent markers into Th1, Th2, Tregs, Bregs, Th17, Th22 and pDCs. Finally, serum cytokines were measured in circulation by luminometry.

Several variables, including serum cytokine content and a complete blood count evaluated by fluorescence-activated cell sorting (FACS), were assessed to obtain an immunological overview of achalasia (Tables 3, 4; Fig. 1). Fluorescence-activated cell sorting, immunohistochemistry, and serum cytokine measurements are complementary techniques that provide insights into the number of cells in circulation or infiltrating affected tissues, as well as the levels of cytokines released into the bloodstream (Table 5). For instance, a specific type of immune cell may exhibit elevated levels in the systemic circulation without necessarily infiltrating the affected tissue, while another immune cell type might be highly concentrated in the tissue but not present in significant quantities in the circulation (Table 6). This indicates diapedesis and tissue-specific infiltration. Immunohistochemistry was utilized to identify local biomarkers within the resected tissue following surgical treatments (Table 7). Not all patients in the clinical management group qualified for all follow-up investigations. Consequently, sample sizes for routine procedures were substantial, while data for non-standard procedures were limited. Due to this constraint, complete blood count measurements were divided into four control groups based on age and sex, while the two achalasia groups were categorized by sex alone. Circulating white blood cell subsets were analyzed via flow cytometry, leading to the creation of two control and two achalasia groups, differentiated by sex. In the case of serum measures, they allowed for a division between healthy subjects and achalasia patients. The concentration of hemoglobin, the width of the distribution of red blood cells, and the number of platelets were measured. Both male and female achalasia patients showed lower platelet counts than younger and older healthy controls. Only male achalasia patients exhibited an increase in the width of the distribution of red blood cells, but without alterations in hemoglobin levels (Table 3).

**Table 5:** Serum cytokines concentrations.

	Healthy donors (n = 500)	Achalasia (all) (n = 189)	Type I (n = 59)	Type II (n = 123)	Type III (n = 7)
Serum interleukins					
IL-1$\beta$ , mean ± SD	13 ± 3	37 ± 13	32 ± 17	38 ± 18	42 ± 20
Median	1	1	1	1	17.3
Range	1.00–64.1	1.00–1002	1.00–324	1.00–1002	1.00–131
IL-6, mean ± SD	30 ± 5	49 ± 10	21 ± 5	40 ± 9	148 ± 45
Median	13.8	22.6	16.6	21.4	115
Range	1.42–156	1.42–354	1.95–76.0	1.42–354	35.0–324
IL-10, mean ± SD	9 ± 2	23 ± 3	30 ± 11	23 ± 3	9.8 ± 0.5
Median	7.1	11.4	15.9	11.6	9.5
Range	0.90–28.0	0.32–105	2.39–105	0.32–74.2	8.00–11.6
IL-17A mean ± SD	32 ± 8	120 ± 40	59 ± 16	109 ± 60	220 ± 51
Median	24	30.7	51	17.4	300
Range	0.23–137	2.86–979	20.9–106	2.86–979	57.3–303
IL-25 mean ± SD	7 ± 4	25 ± 9	26 ± 16	13 ± 4	13 ± 83
Median	1	1	1	1	1
Range	1.00–105	1.00–506	1.00–248	1.00–166	1.00–506
TNF-$\alpha$ mean ± SD	2.3 ± 0.3	3.7 ± 0.4	4.5 ± 1.1	3.2 ± 0.4	6.3 ± 1.4
Median	2.15	2.4	2.73	1.87	6
Range	2.10–7.46	0.2–19.8	0.8–19.8	0.2–14.1	1.4–11.8

**Figure 2:**
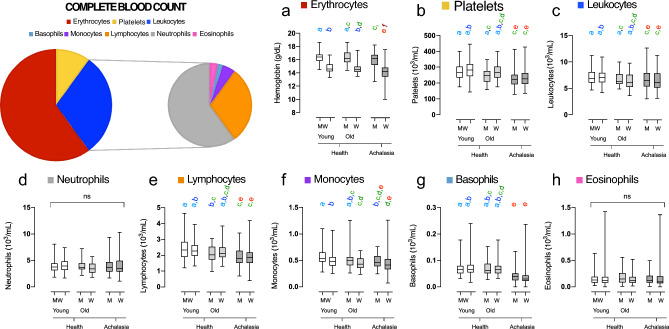
Differences in blood count composition between healthy participants and achalasia patients. Box and whisker plots are used to display the median, interquartile range, and range for hemoglobin concentration and all cellular blood components, including leukocytes includes neutrophils, lymphocytes, monocytes, basophils, and eosinophils. Statistical significance is summarized by levels and represented by colored letters (a–e), where groups with the same letter have comparable significance levels.

**Figure 3:**
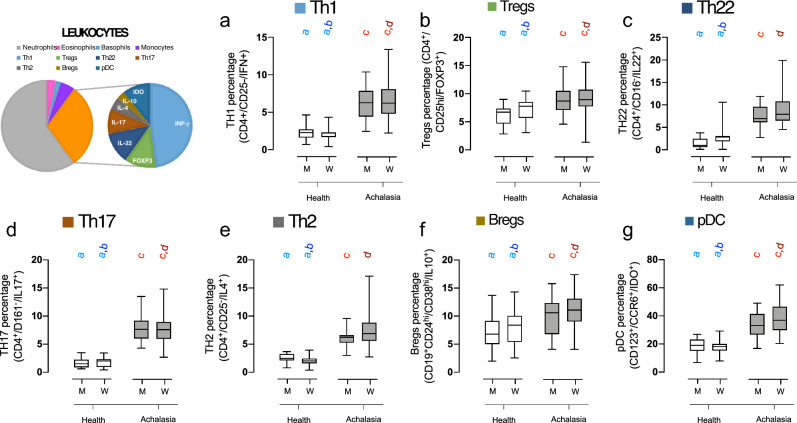
Differences in lymphocyte subtypes between healthy individuals and patients with achalasia. Box and whisker plots show the median, interquartile range, and range of the percentages of fluorescence-activated cell sorting of lymphocytes in men and women with and without achalasia, identified by biomarkers. Statistical significance is summarized by levels and represented by colored letters a-d, where groups with the same letter have comparable significance levels.

**Figure 4:**
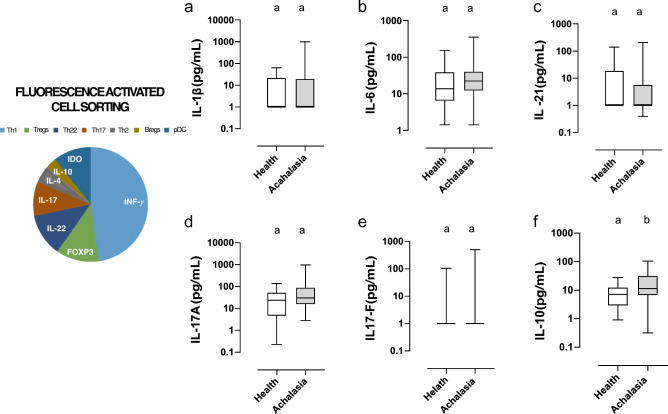
Cytokine serum concentration in healthy controls and achalasia patients. Box and whisker plots illustrate the median, interquartile range, and range of serum cytokine concentrations. Statistical significance is summarized by levels and represented by colored letters a,b, where groups with the same letter have comparable significance levels.

As for the total white blood cell count, no significant differences were observed between groups, except for a slight decrease in the number of leukocytes in female patients with achalasia compared to their healthy counterparts (Fig. 2). Cell counts of neutrophils and eosinophils were similar. A decline in the number of monocytes was observed with increasing age, and among the groups, young males and females exhibited the highest monocyte counts. Both basophil and lymphocyte cell counts were decreased in both men and women with achalasia, yet there was no significant difference in the neutrophil-to-leukocyte ratio (NLR). Flow cytometry was used to divide lymphocytes into distinct subsets. Patients with achalasia had increased circulating CD4+ T cells, CD25- T cells, CD161+ T cells, FOXP3+ Tregs, IDO-expressing regulatory plasmacytoid dendritic cells (pDCregs), and IL-10-expressing regulatory B cells (Bregs) compared to healthy individuals (Fig. 3). Finally, only IL-10 was significantly increased in achalasia patients (Fig. 4).

### Lymphocyte response as the main contributor to the immune response in achalasia

**Figure 5:**
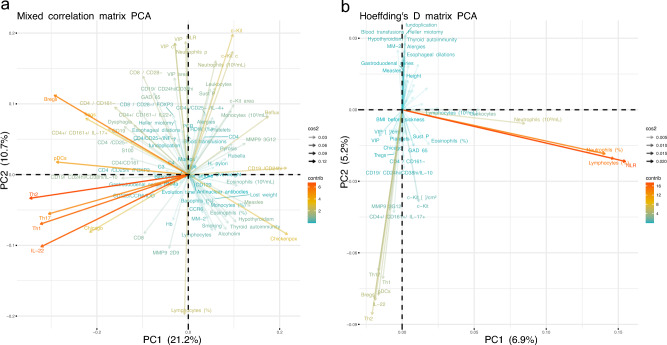
Principal component analysis (PCA) loading plot. (**a**) PCA was performed on the mixed correlation matrix of the complete dataset, normalized by health thresholds of men and women with achalasia. (**b**) PCA was also applied to the Hoeffding’s D statistical dependence matrix. The color of the vector represents the contribution to the first and second principal components, where variables that highly correlated with the principal components contributed the most. The opacity of the vector shows the $\cos ^2$, the rotation of the vector, and the quality of the representation of the variable on the map.

We have previously hypothesized that achalasia is an autoimmune disease^2^. Therefore, the strategy of the present contribution included the collection of a variety of immune-related physiological data from local (Table 6) and systemic tissues (Table 6). Applying principal component analysis (PCA) to the data as in previous contributions^24–26^, we identified the most important variables contributing to the first two principal components (PC1 and PC2), i.e., those two mutually perpendicular vectors that capture the majority of variance within the sample. This method allowed us to identify how white blood cell subpopulations (Th1, Th2, Th17, Tregs, Bregs and pDCregs) and cytokines (IL-22) relate to achalasia (Fig. 5). The second component PC2 is centered on the detection and quantitative assessment of interstitial cells of Cajal (c-Kit+ cells), ganglion cells (VIP+), gelatinase MMP-9-2D9+ cells, as well as the percentages of circulating lymphocytes and neutrophils within the tissue (Fig. 5). Moreover, we adjusted the previous method to increase the readability of the data structure. We utilized Hoeffding’s D, a measure of statistical dependency, instead of correlation between pairs of variables. Whereas correlations can be positive or negative, statistical dependence tends to be positive. As a result, variables were clustered more closely. Neutrophils and lymphocytes contributed significantly to PC2, albeit in opposite ways. Consequently, these measures are the same component when statistical dependence is assessed. Their contributions to the whole dataset became evident and prominent, and consequently they are positioned along the first component (Fig.  5). Additionally, we observed that subsets of Th1, Th2, Th17, IL-22, Bregs, pDCregs, and the cell number of MMP-9-3G12 positive cells, interstitial cells of Cajal, and ganglion cells are linked. Variables regarding the clinical history of the patients are now prominent and shown along the second component. In conclusion, the data-driven strategy separated the most significant contributions from the data structure into three categories: first, neutrophil and lymphocyte variables of the complete blood count; second, variables related to circulating cell subsets, serum cytokines, and tissue biomarkers; and third, clinical data, including fundoplication and Heller myotomy.

### Topology of physiological networks differs between men and women with achalasia

**Figure 6:**
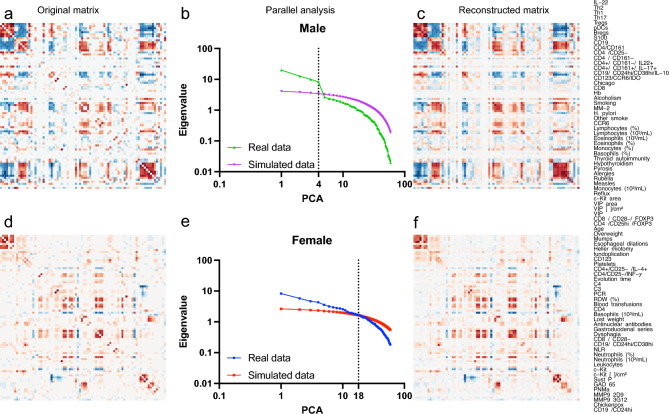
Reconstruction of the correlation matrix using PCA. The left column displays the original correlation matrix for men with achalasia (**a**) and women with achalasia (**d**). The central column shows the decreasing eigenvalue of each component and its comparison to simulated data from a random matrix for men (**b**) and women (**e**). The right column shows the reconstructed correlation matrix employing only the principal components selected by parallel analysis, four for men (**c**) and eighteen for women (**f**). All matrices are arranged following the order depicted on the right. The order of the physiological variables was chosen by optimal leaf order, a hierarchical arrangement that maximizes the sum of the similarities of adjacent leaves in the ordering.

**Figure 7:**
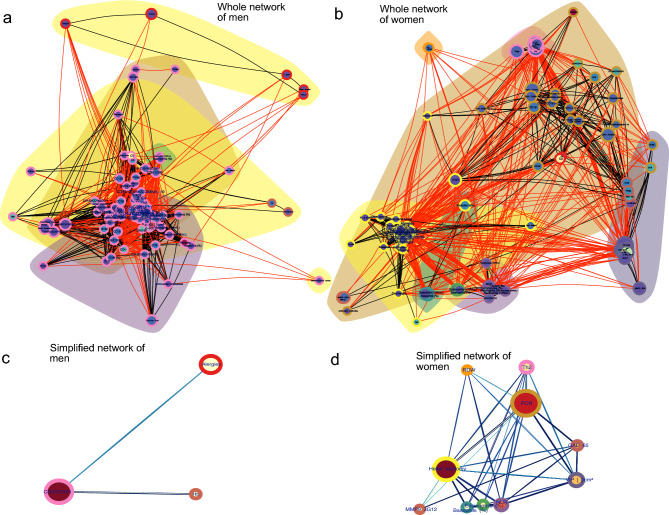
Men and women with achalasia have different physiological networks. The physiological networks for men (**a**) and women (**b**) with achalasia are shown. Colored clouds represent clusters formed by the Louvain algorithm. The border color of a node denotes its cluster according to the InfoMAP algorithm. For intracluster correlations, the edge color is black, while for intercluster correlations, it is red. The node size shows the normalized median and its size shows the alpha value, i.e. the distance from a normal distribution. To simplify the networks of men and women, nodes within the same InfoMAP cluster were collapsed into the most central node location. This highlights the major physiological processes contained within the physiological network for men (**c**) and women (**d**). In these networks, the node size shows the laplacian centrality of the node and its color shows the fractional flow betweenness. Bigger nodes are more influential, while redder nodes are more medial.

**Figure 8:**
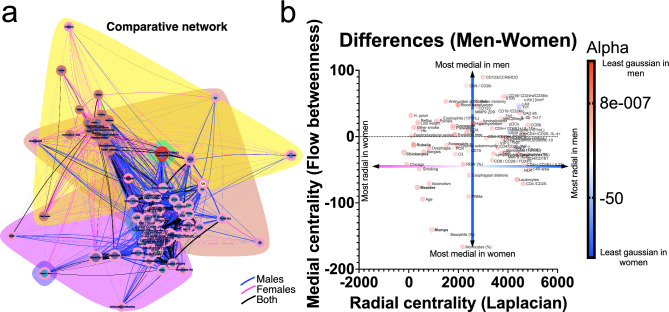
Sexual differences in patients with achalasia. Networks from both sexes were superimposed, with correlations unique to women in pink, correlations only for men in blue, and correlations shared by both sexes in black (**a**). Clouds of color represent Louvain’s optimization clustering and node borders represent InfoMAP’s clustering. Differences in medial and radial node centrality are plotted (**b**). Node size shows the normalized value median and its size shows the alpha value, i.e. the distance from a normal distribution.

Given the physiological distinctions between men and women^25^, particularly in their immunological responses, we conducted separate analyses for each sex. This division remains clear in the correlation matrix of patients with achalasia (Fig.  6). Men need only four principal components to summarize and reconstruct the matrix, whereas women need at least eighteen components (Fig.  6). The difference in the number of necessary components associated with the modularity of each matrix highlights a significant distinction: correlations between variables in men are notably distinct and independent, contrasting with those in women. It is worth noting that the majority of the physiological characteristics obtained for this study are related to the immune system. Consequently, we primarily observe the behavior of the immunological variables. According to a network analysis of the correlations, men and women exhibit distinct interactions (Fig.  7). To build clusters of physiological variables, we employed three different methods: first, we established topological clusters using an energy-model layout in which node position is governed by edge density; a second technique involved optimizing a modularity function to generate node-containing clouds; and a third method implied optimizing the description length of a random walker, an information criterion, to color the border of each node. Similarly to what was found by PCA, physiological networks in men may be summarized by only three nodes while in women ten nodes are necessary (Fig.  7C,D). As shown for other datasets^25,26^, the network for women has a higher edge density (Fig.  7). Some associations appear confined to a specific sex when searching for specific distinctions between the sexes. For instance, biomass exposure and eosinophil concentration correlate with clinical presentation only in females (Fig.  8), while immunological factors in males appear to be more strongly interconnected.

**Table 6:** Tissue cell populations.

	Healthy donors (n = 500)	Achalasia (all) (n = 189)	Type I (n = 59)	Type II (n = 123)	Type III (n = 7)
Tissue cell populations					
IL-22-expressing cells (%) mean ± SD	3 ± 1	14 ± 7	7 ± 4	15 ± 4	29 ± 6
Median	3	15	6	16	28
Range	2.0–5.5	4.0–35.0	4.0–18.0	7.0–22.0	21.0–35.0
IL-17A-expressing CD4 T cells (%) mean ± SD	1.1 ± 0.9	12 ± 5	12 ± 3	8 ± 1	19 ± 3
Median	1	10.3	12	8	19
Range	0.0–2.0	7.0–22.0	9.0–19.5	7.0–11.0	16.0–22.0
IL-4-expressing CD4 T cells (%) mean ± SD	1.6 ± 0.7	7 ± 6	4 ± 1	3.5 ± 0.6	17.8 ± 0.8
Median	1.5	4	4	3.5	18
Range	1.0–3.0	2.0–19.0	3.0–5.0	2.0–4.0	17.0–19.0
IFN-$\gamma$-expressing CD4 T cells (%) mean ± SD	2 ± 1	9 ± 7	14 ± 2	4.8 ± 0.5	18 ± 6
Median	1.5	5.7	13	6	15
Range	0.5–3.5	3.5–29.0	12.5–16.0	3.5–6.0	13.0–29.0
Foxp3-expressing CD25 T cells (%) mean ± SD	4.4 ± 0.6	7 ± 3	6 ± 3	7 ± 3	10 ± 2
Median	4.5	8	7.5	7.5	10
Range	3.5–5.0	3.0–13.5	2.3–9.0	2.0–13.5	8.0–13.0
IL-10-expressing CD20 B cells (%) mean ± SD	1.7 ± 0.5	6 ± 3	14 ± 7	4.5 ± 0.8	10 ± 2
Median	1.5	4.5	15	4.5	10
Range	1.0–2.5	3.0–12.0	4.0–35.0	3.5–6.5	7.0–12.0
IDO-expressing CD123 cells (%) mean ± SD	3.8 ± 0.7	7 ± 2	3.7 ± 0.6	3.7 ± 0.6	10 ± 2
Median	3.8	6	4	4	10
Range	3.0–4.0	4.0–13.0	3.0–4.0	3.0–4.0	9.0–13.0
GAD65-expressing cells (%) mean ± SD	0.3 ± 1.3	39 ± 22	44 ± 19	31 ± 10	70 ± 12
Median	0	33.2	44.3	32	68.3
Range	0.0–5.3	6.3–83.0	16.0–67.0	6.3–63.3	58.7–83.0
PNMa/Ta2-expressing cells (%) mean ± SD	0.3 ± 1.1	24 ± 12	22 ± 8	22 ± 5	47 ± 20
Median	0	21.7	12.6	19.9	43.3
Range	0.0–4.3	0.0–74.7	0.0–74.7	0.0–60.7	28.7–68.7
S100-expressing cells (%) mean ± SD	9 ± 7	22 ± 4	20 ± 4	20 ± 4	52 ± 20
Median	10	18	18	13.3	55.7
Range	0.0–19.3	0.0–69.7	0.0–51.3	0.0–54.0	30.3–69.7
P Substance-expressing cells (%) mean ± SD	1 ± 2	1.7 ± 0.5	2.2 ± 0.7	1.6 ± 0.9	0.6 ± 0.4
Median	0.2	0.6	1	0	0.3
Range	0.0–7.3	0.0–16.3	0.0–7.7	0.0–16.3	0.3–1.0
VIP cells (ganglion cells)/cm^2^ (%) mean ± SD	60 ± 21	1.4 ± 0.6	0.9 ± 0.4	0.4 ± 0.4	0.0 ± 0.0
Median	55.9	1	0	0	0
Range	35.0–105.3	1.0–14.0	0.0–4.0	0.0–7.8	0.0–0.0
Interstitial cells of Cajal (c-Kit)/cm^2^(%) mean ± SD	338 ± 144	96 ± 22	127 ± 42	98 ± 29	4 ± 4
Median	314.7	52.2	71.5	49	5
Range	190.9–776.2	0.0–608.4	7.0–608.4	7.8–479.0	0.0–7.0
MMP-9 3G12-expressing cells (%) mean ± SD	4 ± 2	11 ± 9	14 ± 10	11 ± 1	5 ± 2
Median	4.2	9.8	14.5	9.5	6.2
Range	0.0–5.7	0.7–4.7	4.3–46.3	0.7–40.7	3.0–6.3
MMP-9 2D9-expressing cells (%) mean ± SD	0.4 ± 0.9	0.9 ± 0.2	0.7 ± 0.3	1.2 ± 0.4	0.8 ± 0.2
Median	0	0.3	0.2	0.3	0.7
Range	0.0–3.0	0.0–4.7	0.0–4.3	0.0–4.7	0.7–1.0

**Table 7:** Autoantibodies presence.

	Healthy donors (n = 500)	Achalasia (all) (n = 189)	Type I (n = 59)	Type II (n = 123)	Type III (n = 7)
Auto-antibodies					
GAD65, (%)	NA	83	89	80	80
PNMA2, (%)	NA	90	89	87	100
Recovering, (%)	NA	0	0	0	0
Yo, (%)	NA	0	0	0	0
Zic4, (%)	NA	7	0	13	0
Titin, (%)	NA	3	0	7	20
SOX1, (%)	NA	3	11	7	0
Hu, (%)	NA	3	11	0	0
Ri, (%)	NA	3	11	0	0
Amphiphysin, (%)	NA	3	0	0	20
Antinuclear antibodies n, (%)	NA	78 (41)	19 (32)	54 (44)	5 (71)

## Discussion

Idiopathic achalasia is an archetypal esophageal motor disorder caused by persistent inflammation at the Auerbach plexus, resulting from a loss of inhibitory neurons of the myenteric esophageal plexus. The etio- and physiopathogenic mechanism for achalasia is thought to involve a repetitive insult from a neurotropic infectious agent. Progression to the disease only occurs in infected individuals with a genetic predisposition for an aggressive, chronic inflammatory response. In most cases, pathology is accompanied by neuronal antibodies that contribute to the destruction of the myenteric plexus. For achalasia, multiple reports are associated with autoimmune diseases such as polyglandular autoimmune syndrome Type II^27^,thyroid disease^28^, vitiligo^29^, and common variable immunodeficiency^30^. In previous studies, and also the present contribution, several systemic biomarkers of immune response were detected at higher levels in patients with achalasia (Fig. 5), particularly those involved with white blood cell subtypes. Nonetheless, these biomarkers encompass various physiological systems, including age, sex, innate and adaptive immune cell subsets, autoantibodies, clinical characteristics, etc. This growing array of available biomarkers lacks an explanatory framework on how and why homeostasis becomes altered by achalasia. Using PCA, we showed that these systemic biomarkers are the main drivers of achalasia (Fig. 5), inducing the variance of the physiological measures of the patients. Moreover, we showed how, exclusively for women, the history of previous childhood exanthemata infections (mumps, measles, chickenpox and rubella) is a leading influencer and mediator within the physiological network (Fig. 8). Indeed, even if traditional (univariate) statistical approaches fail to detect differences in average values between groups for a variety of immunological variables, there are notable differences in how these variables are positioned within the corresponding (multivariate) network (Fig. 8). It is not preposterous to propose a viral etiology in achalasia because previous studies have established a link between viral infection and the trigger of the disease. For example, a longitudinal analysis has shown compellingly that Epstein–Barr virus infection is the main trigger of multiple sclerosis^31^,an autoimmune disease that until the last decade had no clear cause. An important environmental factor must be deduced for some autoimmune diseases due to a high percentage of disease-discordant monozygotic twin pairs^32^.There are several theoretical mechanisms by which infections may act as triggers of autoimmunity^32–34^.

In the present study, it was found that men and women with achalasia were equally infected in childhood by exanthemata viral infections, and there were no meaningful differences in immune physiological variables between sexes. However, these diseases had a significant contribution to the immune landscape in which achalasia occurs. This suggests that, although no single viral infection may be responsible for achalasia, vaccination against preventable diseases is a priority for women. One limitation of the study is its cross-sectional nature, which makes it difficult to deduce causal connections. The biomarkers employed were chosen based on their availability and accessibility. Another significant limitation of the study is that some measures, such as FACS, are expensive and therefore not widely available for clinical practice, while others, such as a biopsy of the LES, cannot be justified in healthy subjects. Despite these limitations, the correlational approach enabled us to show how physiological variables behave differently in each group. Sex-dependent differences in network topology^25^ may determine how the disease presents and develops. The male physiological network is more adaptable and modular, making it more vulnerable to pathologies that attack multiple systems simultaneously, such as COVID-19^26^. In contrast, female networks are more robust and have multiple inter-cluster links, making them more likely to spread out the impact of localized diseases (Figs. 7, 8).

### Conclusion

From a network perspective, it is possible that the robust (densely connected) physiological network of women makes them more susceptible to long-term immune changes after various childhood viral exanthems, while these viral exanthems are not as influential in the modular network of men. Prevention of viral infections must be prioritized as part of medical care particularly, in women. Further longitudinal population studies are required to determine whether changes in several viral infections reflect incidence changes in achalasia.

## Methods

### Design

This was an exploratory, observational, and cross-sectional study conducted at a tertiary referral care center (Instituto Nacional de Ciencias Médicas y Nutrición Salvador Zubirán) between April 2015 and December 2021. Participants included 189 consecutive patients with idiopathic achalasia (Type I: n = 58; Type II: n = 124; and Type III: n = 7) and 500 healthy control donors. Not all medical procedures are justified for healthy control donors, hence tissue biopsies, flow cytometry and auto-antibody determination were not performed extensively in the control group.

### Patients

All patients were diagnosed by high-resolution manometry (HRM), upper endoscopy, and esophagogram. Patients aged $\ge$ 18 years were enrolled in the study. Exclusion criteria included Chagas disease, esophageal stricture, esophageal scleroderma, gastric or esophageal cancer, peptic stricture, other esophageal motility disorders, pregnancy, hematologic disease, cancer, severe renal or liver disease, ongoing infection, or use of aspirin or steroid treatment. Clinical records of patients were carefully reviewed according to a pre-established protocol. Data were collected retrospectively for each study participant from the medical records of the hospital, including demographic features, type of achalasia, family history of autoimmunity, and current diagnosis of organ or systemic autoimmunity. When a concurrent autoimmune diagnosis was identified, we extracted all pertinent information, including the date of diagnosis, symptoms at the time of diagnosis, clinical and laboratory confirmatory test results, and administered treatment. Finally, chronic inflammatory conditions (i.e., asthma, allergic rhinitis, gout, and rosacea) were also extracted. Complete blood count (CBC) parameters were the latest laboratory findings recorded before surgical intervention. For comparison, 500 healthy controls who volunteered at the blood bank were recruited for the study to obtain healthy CBC parameters (Table 1). None of the included controls had cardiovascular, metabolic, inflammatory, or neoplastic disease. Demographic, clinical, and laboratory information were also collected.

### Laboratory information

All CBC analyses were performed with an automatic hematologic analyzer (Beckmancoulter DxH 800 Hematology Analyzer). Hemoglobin (Hb), white blood cell (WBC), neutrophils, lymphocytes, monocytes, eosinophils, and platelet counts were obtained before surgical treatment. Blood samples were collected in di-potassium ethylenediaminetetraacetic acid tubes.

### Flow cytometry

Peripheral blood mononuclear cells (PBMCs) were obtained by gradient centrifugation on Lymphoprep (Axis-Shield PoC AS, Oslo, Norway). The cell pellet was resuspended in 1 mL RPMI at 1–2 $\times 10^{6}$ cell/mL. The cell suspension was treated with 2 $\upmu$L of a cell activation cocktail of phorbol-12myristate 13-acetate (40.5 mM) and ionomycin (669.3 mM) in DMSO (500$\times$) and brefeldin A (BioLegend Inc., San Diego, CA) for 6 h at 37 ^∘^C in a CO2 incubator. PBMCs were incubated with 5 mL of Human TruStain FcXTM (BioLegend Inc.) per million cells in 100 mL PBS for 10 min. Then they were labeled with 5 mL of anti-human CD3-FITC-labeled, anti-human CD4-PeCy5-labeled, and anti-human CD161- APC-conjugated monoclonal antibodies (BD Biosciences, San Jose, CA); anti-human CD3-FITC-labeled, anti-human CD4-PeCy5-labeled and anti-human CD25-APC-conjugated monoclonal antibodies (BD Biosciences) in separated tubes during 20 min at 37 ^∘^C in the dark. Cells were permeabilized with 200 mL of cytofix/cytoperm solution (BD Biosciences) at 4 ^∘^C for 30 min. Intracellular staining was performed with an anti-human IL-22-PE-, IL-17A-PE-, IL-4-PE-, IFN-$\gamma$-PE-labeled mouse monoclonal antibodies (BD Biosciences) for 30 min at 4 ^∘^C in the dark. An electronic gate was made for CD3+/CD4+/CD161- cells, CD3+/CD4+/CD161+ cells, and CD3+/CD4+/CD25-cells (Fig. 1). Results are expressed as the relative percentage of IL-22+, IL-17A+, IL-4+, and IFN-g+ expressing cells in each gate (Fig. 2). As isotype control, IgG1-FITC/IgG1-PE/CD45-PeCy5 mouse IgG1 kappa (BD Tritest, BD Biosciences) was used to set the threshold and gates in the cytometer. We ran an unstained (autofluorescence control) and permeabilized PBMCs sample. Autofluorescence control was compared to single-stained cell positive controls to confirm that the stained cells were within the normal range for each parameter. Additionally, BD Calibrate 3 beads were used to adjust instrument settings, set fluorescence compensation, and check instrument sensitivity (BD calibrates, BD Biosciences). Fluorescence minus one (FMO) control was stained in parallel using the panel of antibodies with the sequential omission of one antibody, except for the anti-IL-22, anti-IL-17A, anti-IL-4, anti-IFN-$\gamma$, which was replaced by an isotype control rather than omitted. Finally, T subsets were analyzed by flow cytometry with an Accuri C6 (BD Biosciences). 500,000 to 1,000,000 events were recorded for each sample and analyzed with the FlowJo X software (Tree Star, Inc.).

### Serum collection

All cytokines, hsCRP, antinuclear antibodies (ANAs), and anti-myenteric autoantibodies were measured by serum isolated from peripheral blood samples. Blood samples were taken from healthy donors and from patients with achalasia without treatment and before the start of surgical procedures. Samples were collected in serum separator tubes, followed by five tube inversions. After allowing the samples to stand for 20 min, they were centrifuged at 2000 rpm at 4 ^∘^C. The supernatants were aspirated and separated into 300 $\upmu$l aliquots, and then stored at − 70 ^∘^C.

### Antinuclear antibodies (ANA) testing

ANA assessment was performed by indirect immunofluorescence with HEp-2 cells IgG isotype (Inova Diagnostics Inc, San Diego, CA). Positivity was assigned according to our local cut-off values^35^ (i.e., speckled:> 1:160; nucleolar:> 1:40; cytoplasmic:> 1:40; mitochondrial:> 1:160; and others: > 1:40).

### Immunoblot analysis

To analyze the presence and specific target antigens of 12 circulating anti-myenteric autoantibodies, sera were tested with the EUROLINE paraneoplastic neurologic syndromes 12 Ag (IgG) qualitative kit (Euroimmun AG, Lübeck, Germany). This test is a membrane strip coated with parallel lines of a highly purified combination of neuronal antigens, including amphiphysin (antibodies against amphiphysin, a synaptic protein), CV2 (anti-66 kDa protein antibodies), and PNMA2 (proteins in the nucleoli of the neuronal cell nuclei, Ma2/Ta), as well as onconeural antigens, such as Ri (anti-neuronal nuclear antibodies-2, ANNA-2), Yo (anti-Purkinje cell autoantibodies, PCA-1), Hu (anti-neuronal nuclear antibodies-1, ANNA-1), recoverin (anti-23kDa and 65 kDa recoverin), SOX-1 (anti-glia nuclear antibodies, AGNA), titin, zic4, GAD65 (antibodies against the enzyme glutamic acid decarboxylase) and Tr (autoantibodies against Tr, a protein in the cytoplasm of cerebellar Purkinje cells, DNER). After blot strip blocking, sera of patients and positive controls (IgG) were incubated at 1/100 dilution for 1 h at room temperature on a rocking shaker. To detect the bound antibodies, a second incubation was carried out using alkaline phosphatase-labeled anti-human IgG for 30 min at room temperature on a rocking shaker, and the color reaction was revealed with a substrate solution (nitroblue tetrazolium chloride/5-Bromo-4-chloro-3-indolyl phosphate). The interpretation of the results was performed using the EUROLine Scan software (Euroimmun).

### Determination of cytokine concentration by Bioplex

The concentrations of IL-1$\beta$, IL-6, IL-10, IL-17A, IL-17F, IL-22, IL-23, IL-33, IFN-$\gamma$, and TNF-$\alpha$ were measured using commercial Multiplex kits (ThermoFisher) according to the instructions of the manufacturer.

### Tissue biopsies

Biopsies of the lower esophageal sphincter (LES) muscle were taken from achalasia patients during Heller myotomy (HM). After completion of the myotomy without using energy devices, a full-thickness muscle biopsy (2 mm wide and 2 cm long) was obtained by cutting with scissors and was immediately preserved in formalin. LES samples of transplant donors were included as non-inflamed tissue controls. The esophagogastric junction was obtained during organ procuration, with previous signed informed consent from the family. The esophagogastric junction was taken with 3 cm of the esophagus and 2 cm of the stomach. The tissue was transported at 4 ^∘^C in Bretschneider’s (Custodiol) solution for a period of maximally 4–6 h. Subsequently, a full-thickness biopsy of the esophagus muscle (including the myenteric plexus) was obtained. Tissue was immediately formalin-fixed and paraffin-embedded.

### Immunohistochemistry

4 $\upmu$m thick tissue sections were deparaffinized and rehydrated. Then heat-mediated antigen retrieval with citrate buffer pH 6.0 or EDTA buffer pH 8.0 was performed. Tissues were incubated for 18 h at 4^∘^C with mouse monoclonal or rabbit polyclonal primary antibodies diluted according to the recommendations of the manufacturer in a humidified chamber (Table 8). Binding was detected by incubating sections with a biotinylated donkey or goat anti-mouse or rabbit IgG antibody (ABC Staining System; Santa Cruz Biotechnology). Slides were incubated with horseradish peroxidase- (HRP-) streptavidin and 3,3$\prime$-diaminobenzidine (DAB) (Sigma-Aldrich) or alkaline phosphatase and aminoethyl carbazole and counterstained with Mayer’s hematoxylin (Lillies’ modification). Negative control stainings were performed with normal human serum diluted at 1:100, instead of with primary antibody, and with the IHC universal negative control reagent specifically designed to work with rabbit, mouse, and goat antibodies (IHC universal negative control reagent, Enzo Life Sciences, Inc., Farmingdale, NY, USA). The reactive blank was incubated with phosphate buffer three saline-egg albumin (Sigma-Aldrich) instead of the primary antibody. Control experiments excluded the presence of nonspecific staining or endogenous enzymatic activities. At least two different sections and two fields were examined for each biopsy. Results are expressed as the mean ± standard error of the mean (SEM) cells quantified by Image-Pro Plus version 5.1.1. To detect gelatinase B/MMP-9 proteoforms, we used two monoclonal antibodies against human MMP-9.9 REGA-2D9 that recognizes the inactive human pro-MMP-9, whereas REGA-3G12 recognizes the activated proteoform lacking the propeptide. Previously we demonstrated that REGA-3G12 neutralizes the activity of gelatinase B/MMP-9 in vivo. Immunoreactivity with REGA-3G12 constitutes a proxy for activated MMP-9 and in situ gelatinase B activity distinguished from the immunoreactivity with REGA-2D9, the latter of which reveals the presence of inactive pro-MMP-9.

**Table 8:** Antibodies for immunohistochemistry.

Antibody	Origin	Antigen retrieval	Dilution	Origin
VIP	Mouse monoclonal	Citrate buffer of pH 6	1:150	Santa Cruz Biotechnology,
c-kit (CD117)	Rabbit monoclonal	Citrate buffer of pH 6	1:500	BioSB, Santa Barbara, CA
GAD65	Rabbit polyclonal	Citrate buffer of pH 6	1:100	Abcam
PNMa	Rabbit polyclonal	EDTA buffer of pH 8	1:100	Abcam
S100	Mouse monoclonal	Citrate buffer of pH 6	1:3000	Abcam
P Substance	Mouse monoclonal	Citrate buffer of pH 6	1:3000	Abcam
REGA-3G12	Mouse monoclonal	Citrate buffer of pH 6	1:100	Rega Institute
REGA-2D9	Mouse monoclonal	Citrate buffer of pH 6	1:100	Rega Institute

### Ganglion cells and myenteric interstitial cells of Cajal analysis

Morphometric analysis of ganglion cells (VIP+ cells) and interstitial cells of Cajal (CD117+ or cKit+ cells) in patients and controls was performed in 5–10 photographs at a magnification of 40$\times$. Cells were quantified with Image-Pro Express v6.3 (Media Cybernetics Inc.). The area of each measurement was 3.1 $\times$ 105 $\upmu$m^2^ (0.031 cm^2^). The number of cells was obtained using the formula: [total number of cells/number of measured fields] $\times$ 0.028 cm^2^. Results are expressed as mean ± standard error of the number of cells (SEM).

### Statistical analysis

As important differences between men and women may be present, the database was separated by sex^25^. Due to the many variables with non-normal distributions, non-parametric approaches were used for statistical analysis. As a measure of distance from a Gaussian distribution, we used the radius in a space built from the first four distribution moments: average $\mu$, standard deviation $\sigma$, skewness *sk* and kurtosis *k*^36^,

$$\alpha =\sqrt{\left( \frac{\sigma }{\mu } \right) ^{2} + sk^2 + k^2}.$$

This radius increases as the distribution moments of the variable grow further from an ideal Gaussian distribution. These values allowed us to determine whether a variable distribution differs between men and women. For comparing two groups, we used the Mann–Whitney U test with the FDR (Benjamini–Krieger–Yekutieli) approach for multiple comparisons. In contrast, we used the Kruskal–Wallis test with Dunn’s post hoc test for comparing three or more groups. An exact Fisher test was performed to compare the odds ratio of binomial variables. We used Spearman rank correlation to calculate the correlation between two quantitative and continuous variables in the network interaction. Polyserial, polychoric, and tetrachoric correlations were performed appropriately when ordinal and binomial variables were involved, hence this correlation matrix is called a “mixed” correlation matrix. These correlations had values that range from 1, indicating a perfect positive correlation, to 0 for no correlation, to − 1, indicating a perfect negative correlation. To clarify the relationship between variables, we studied the matrix of Hoeffding’s D for all available pairs of variables. The D statistic is robust against a variety of possible deviations from the statistical requirement of independence, including non-monotonic correlations. The value of this statistic may range from 1, indicating a perfect statistical dependency, to 0, indicating no statistical dependence, regardless of whether correlation is positive or negative. However, negative values down to − 0.5 may be found when values are repeated exactly in the dataset. This may happen in variables where continuous values are discretized due to the measurement method, e.g. in blood pressure measurement, where the value is rounded to the closest multiple of ten. Notably, this also happens in ordinal data.

### Principal component analysis (PCA)

PCA was performed afterwards both on the mixed correlation matrix and the matrix of Hoeffding’s D statistical dependence using the mixedCor function of the psych package and the hoeffd function of the Hmisc package respectively. The correlation matrix offers a robust strategy to perform PCA^37^. The parallel analysis compares the scree diagrams of eigenvalues of the empirical correlation matrix to the eigenvalues of a random matrix of the same size as the original data matrix. Principle components (PCs) are considered to be meaningful if its eigenvalue is larger than the corresponding eigenvalue of the simulated data. The empirical correlation matrix may be recreated from the meaningful PCs with minimal information loss. Loading plots of the first and second PCs are shown to identify which variables have the most significant effect.

### Network analysis

Complex Inference Networks is a methodology that allows the construction of networks in which the links are inferred rather than explicitly observed. Here, complex refers to networks with properties that are neither fully random nor completely ordered. Correlation-based networks are a typical and widely used tool for making such inferences. As previously indicated, a mixed correlation matrix for men and women as separate groups was generated^25^. A p value threshold of 0.05 was applied to correlation matrices to build adjacency matrices for constructing physiological networks. The network layout was determined via the Lin-Log energy model for topological clustering. This model dictates the node position by edge density while disregarding path length, which allows for the superposition of nodes with high collinearity^38^. The Louvain modularity optimization algorithm^39^ and the minimal description of a random walker infoMAP clustering algorithm^40^ were executed on the constructed network. Topological and network analyses were performed as described previously in other works for weighted undirected networks constructed from correlation matrices^24^. In brief, node centrality measures and graph-level indices were calculated using the R programming package. Graph-level indices summarize in one quantity a given property that depends on the network, for example, network density, clustering coefficient, characteristic path length, and small-world index. The intuition provided by node centralities in a network can be summarized into radial and medial centralities. A radial measure counts how many pathways have a specific node as an endpoint or “destiny”, while a medial measure counts the number of pathways in which the node acts as an interior point or “bridge”^41^. We chose Laplacian centrality as a metric of the first case, and flow betweenness as a metric of the second case. To show clearly which correlations are different between physiological networks of men and women, we superimposed both networks and colored the sex-exclusive correlations differently. As networks grow in complexity with an increasing number of nodes, we simplified them by consolidating nodes within the same InfoMAP cluster into a single node. This new node was positioned at the original location of the most central node, specifically the one with the highest Laplacian centrality.

### Ethical approval

The studies involving human participants were reviewed and approved by the Ethics Committee from Instituto Nacional de Ciencias Médicas y Nutrición Salvador Zubiran. The patients/participants provided their written informed consent to participate in this study.

## Data Availability

The datasets generated and/or analyzed during the current study are available in the CALMECAC repository at the following web link: https://www.c3.unam.mx/calmecac/. Username: ACHALASIA; Password: Furuzawa.
